# Copine A regulates the size and exocytosis of contractile vacuoles and postlysosomes in *Dictyostelium*

**DOI:** 10.1002/2211-5463.12874

**Published:** 2020-05-19

**Authors:** Elise M. Wight, Amber D. Ide, Cynthia K. Damer

**Affiliations:** ^1^ Biology Department Central Michigan University Mount Pleasant MI USA

**Keywords:** contractile vacuole, copine, *Dictyostelium*, endocytosis, exocytosis, postlysosome

## Abstract

Copines are a family of cytosolic proteins that associate with membranes in a calcium‐dependent manner and are found in many eukaryotic organisms. *Dictyostelium discoideum* has six copine genes (*cpnA*‐*cpnF*), and cells lacking *cpnA*(*cpnA^−^*) have defects in cytokinesis, chemotaxis, adhesion, and development. CpnA has also been shown to associate with the plasma membrane, contractile vacuoles (CV), and organelles of the endolysosomal pathway. Here, we use *cpnA^−^* cells to investigate the role of CpnA in CV function and endocytosis. When placed in water, *cpnA^−^* cells made abnormally large CVs that took longer to expel. Visualization of CVs with the marker protein GFP‐dajumin indicated that *cpnA^−^* cells had fewer CVs that sometimes refilled before complete emptying. In endocytosis assays, *cpnA^−^* cells took up small fluorescent beads by macropinocytosis at rates similar to parental cells. However, *cpnA^−^* cells reached a plateau sooner than parental cells and had less fluorescence at later time points. p80 antibody labeling of postlysosomes (PL) indicated that there were fewer and smaller PLs in *cpnA^−^* cells. In dextran pulse‐chase experiments, the number of PLs peaked earlier in *cpnA^−^* cells, and the PLs did not become as large and disappeared sooner as compared to parental cells. PLs in *cpnA^−^* cells were also shown to have more actin coats, suggesting CpnA may play a role in actin filament disassembly on PL membranes. Overall, these results indicate that CpnA is involved in the regulation of CV size and expulsion, and the maturation, size, and exocytosis of PLs.

## Graphical abstract

The contractile vacuole system in *Dictyostelium* is made up of tubules and bladders that expel water during osmotic stress. *cpnA^−^* cell bladders are large and persistent. Late in the endolysosomal pathway, acidic lysosomes (red) neutralize via an actin coat (red spokes) becoming postlysosomes (PLs) (yellow). Larger, mature PLs are exocytosed. In *cpnA^−^* cells, PLs do not mature and exocytose prematurely.

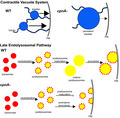

**Abbreviations**
Cpn: copine
CV: contractile vacuole
KO: knockout
PL: postlysosome

Copines are highly conserved calcium‐dependent membrane‐binding proteins characterized by having two C2 domains at the N terminus followed by an A domain at the C terminus [1]. The C2 domain is a calcium‐dependent phospholipid‐binding motif, and many proteins with two C2 domains are involved in membrane trafficking and vesicle fusion [2]. The A domain is similar to the VWA domain in integrins and functions as a protein‐binding motif [3, 4]. Tomsig *et al*. [4] hypothesized that the molecular function of copines was to bind to proteins and shuttle them to membranes in response to a rise in intracellular calcium concentration. Indeed, these authors found that the A domains of human copines interacted with over 20 different proteins with diverse functions, suggesting that copines are involved in the regulation of a multitude of calcium‐dependent cellular processes.

Copines are found in many eukaryotic organisms from *Paramecium* to humans, suggesting thatcopines play a fundamental role in cellular function [1]. Copines have been studied in model organisms and as hypothesized by Tomsig *et al*. [4] are involved in many processes such as growth, development, defense, signaling, cell division, cytoskeletal function, and membrane trafficking [5, 6, 7, 8, 9, 10]. For example, in *Arabidopsis*, the BON1/CPN1 gene was found to have roles in promoting cell growth and development and repressing cell death [5]. In mice, the brain‐specific Copine‐6 was found to be involved in changes to dendritic spine morphology in hippocampal neurons in response to neuronal activity [9].

We are studying the function of copines in the model organism, *Dictyosteliumdiscoideum*, which has six copine genes, *cpnA‐F*, that share 28–60% amino acid identity [11]. We have focused our studies on one of the copines, CpnA. Our studies on *cpnA* knockout (KO) mutants (*cpnA^−^*) in *Dictyostelium* suggest that CpnA is involved in many cellular functions including cytokinesis, adhesion, and chemotaxis, and developmental functions including aggregation, slug phototaxis and thermotaxis, culmination, and stalk cell formation [12, 13, 14, 15]. CpnA binds to membranes in a calcium‐dependent manner and specifically binds to acidic phospholipids with strongest binding to phosphatidylserine and phosphatidylinositol phosphate [11, 16]. GFP‐tagged CpnA was found in the cytoplasm in live cells but translocated to the plasma membrane in response to cAMP stimulation [16]. In fixed cells, CpnA localized to the plasma membrane, endosomes, lysosomes, phagosomes, and contractile vacuoles (CV) [11]. Recently, we have shown that CpnA was able to bind to actin filaments *invitro*, and *cpnA^−^* cells exhibited a reduced actin polymerization response to cAMP stimulation [15]. In this study, we explored how CpnA may functionally contribute to two membrane trafficking systems in *Dictyostelium*: the CV and endolysosomal systems.

Many single‐celled organisms including *Dictyostelium* have a specialized membranous osmoregulatory system, known as the CV system. This system of bladders is interconnected by a network of tubules that allows the cell to respond to osmotic pressure by internalizing and expelling water. The CV bladder and tubule membranes are populated with vacuolar (H^+^)‐ATPases that pump protons into the system’s lumen, which facilitates the influx of water [17]. The bladder fills with water, or charges, and is then targeted to the plasma membrane for expulsion by exocytosis [18]. The acidic CV system is also suggested to be a Ca^2+^ store [19] and that Ca^2+^ released from the CV initiates fusion to the plasma membrane [20].

The endolysosomal system of eukaryotic cells involves the intricate coordination of intracellular membrane‐bound compartments used to internalize, sort, degrade, and recycle material. Molecules are internalized by endocytosis and brought to early endosomes, a mildly acidic pH, and principal sorting organelle. Internalized molecules move through the endolysosomal system from endosomes to late endosomes to lysosomes [21]. Studies with *Dictyostelium* have shown that F‐actin binds to vacuolar (H^+^)‐ATPases of the lysosomal membrane and facilitates the pinching off of membrane vesicles containing the proton pumps [22]. During this process, the lysosome matures into a postlysosome (PL), which has a neutral pH and is nearly twice the size of the lysosome at 2 µm in diameter [23]. The PL then fuses with the plasma membrane [24], which is stimulated by Ca^2+^ [25].

## Materials and methods

### Cell culture

Two *D. discoideum* cell types, NC4A2 and AX4, which we refer to as parental cells, were used. Cells were grown either on Petri dishes or shaking at 180 r.p.m. at 20 °C in HL‐5 media (0.75% proteose peptone, 0.75% thiotone E peptone, 0.5% Oxoid yeast extract, 1% glucose, 2.5 mm Na_2_HPO_4_, and 8.8 mm KH_2_PO_4_, pH 6.5). To prevent bacterial contamination, 60 U·mL^−1^ of penicillin/streptomycin was added to HL‐5. *cpnA* null mutant cells were grown in HL‐5 media supplemented with 10 µg·mL^−1^ blasticidin.

### Creation of *cpnA* null mutants in AX4 cells

**Fig. 1:**
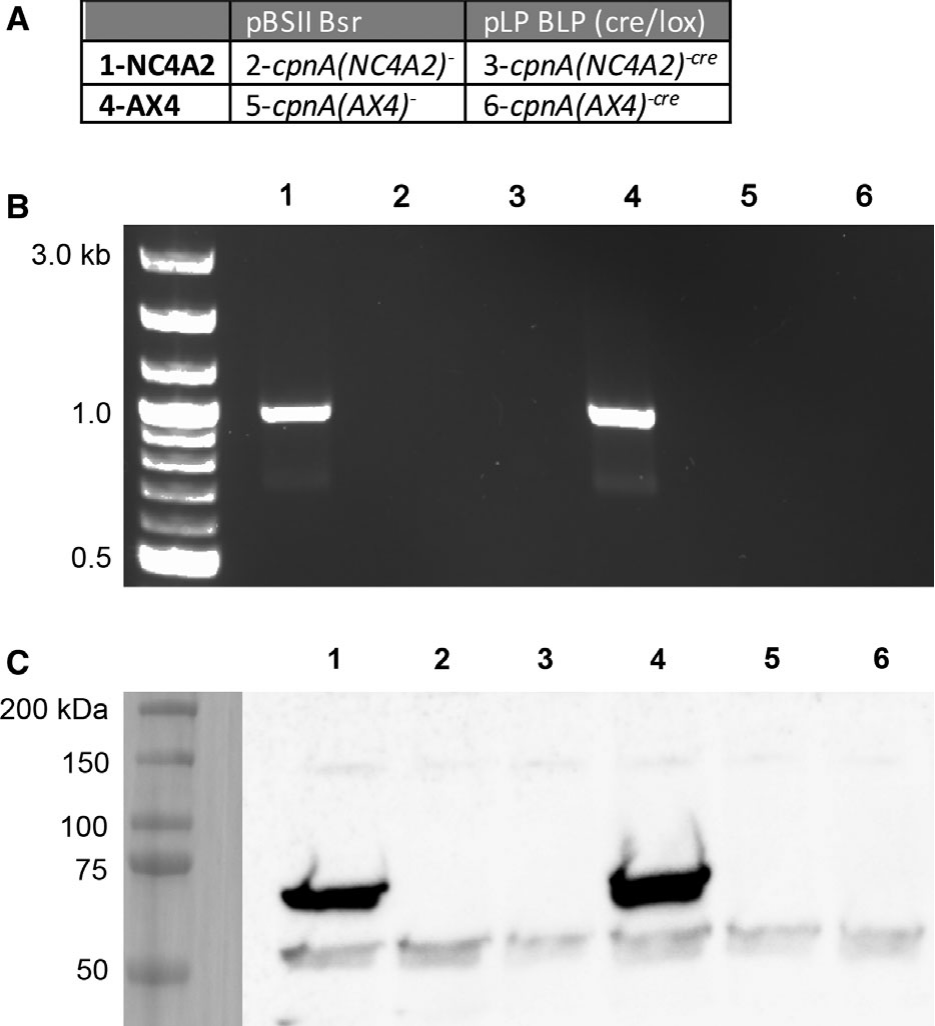
Verification of *cpnA* KO cell lines. (A) Four *cpnA* KO cell lines were made using two different *cpnA* KO plasmids, pBSII Bsr and pLP BLP, in two different axenic strains, NC4A2 and AX4. The table indicates the nomenclature used to refer to the different *cpnA* KO cell lines and the number given corresponds to the numbers in B and C. (B) Genomic DNA was isolated from parental *Dictyostelium* cell lines and cell populations showing resistance to blasticidin after transformations with *cpnA* KO plasmids (pBSII Bsr and pLP BLP). A 1008‐bp region within *cpnA* was amplified by PCR. Cells that lacked an amplification product indicate successful replacement of *cpnA* with *bsr*. (C) Whole‐cell samples (1 × 10^6^ cells) of parental strains and cell populations lacking *cpnA* PCR amplification were analyzed by western blot with CpnA antisera.

Two independent *cpnA* null cell lines were created previously [12, 15] by replacing the *cpnA* gene with the blasticidin resistance gene, *bsr*, in the *Dictyostelium* axenic strain, NC4A2, using two different plasmids: pBSII Bsr [26] and pLP BLP [27]. To create the *cpnA* KO plasmids, PCR fragments of approximately 1 kb upstream (5′) and downstream (3′) of the *cpnA* gene were ligated into the pBSII Bsr plasmid to flank the *bsr* cassette. The *cpnA* gene 5′ and 3′ flanking sequences were removed from the pBSII Bsr plasmid and ligated into pLP BLP on either side of the *bsr* gene, flanked by *loxP* sites. In this study, we used these two different *cpnA* KO constructs to create *cpnA* KO cells using another axenic cell line, AX4. The *cpnA* KO constructs were electroporated into AX4 cells, and cells were plated in 96‐well plates. Transformed cells were selected by resistance to the antibiotic blasticidin (10 µg·mL^−1^) and further screened by western blot and polymerase chain reaction. Whole‐cell samples containing 1 × 10^6^ cells were analyzed by western blot with a CpnA antiserum recognizing the first C2 domain of CpnA [11] at a dilution of 1 : 2000. The CpnA primary antibody was detected by anti‐rabbit IgG peroxidase‐conjugated antibody (1 : 10 000; Sigma, A9169, St. Louis, MO, USA) and chemiluminescence. Polymerase chain reactions amplified the 1008‐bp A domain sequence of *cpnA*. Genomic DNA was extracted in a quick genomic prep [28] by mixing 1 × 10^6^ cells with 25 µL of lysis buffer (50 mm KCl, 10 mm Tris pH 8.3, 2.5 mm MgCl_2_, 0.45% NP40, and 0.45% Tween 20) and 1 µL of proteinase K (20 µg·µL^−1^). Proteinase K was inactivated at 95 °C for 1 min. The genomic DNA was used immediately in a reaction containing Extender™ PCR‐to‐Gel Master Mix (Amresco, N867, Solon, OH, USA) and primers 5′‐GCTCGAGCAGTAATTGAGAGAGAATATAAC‐3′ and 5′‐GGTTATTAATTCTTTTCTAATGATACATTTG‐3′. PCR products were separated on a 1% agarose gel containing 0.5 µg·mL^−1^ ethidium bromide. The *cpnA* KO cell lines were named based on the parental strain and whether the pBSII or pLP BLP plasmid was used (Fig. 1A).

### Contractile vacuole imaging

To image CVs, parental NC4A2 and AX4 cells and their respective *cpnA* KO cells were plated on glass‐bottom dishes (MatTek, 35‐mm dish, 1.5 coverslip, Ashland, MA, USA). After allowing the cells to settle for 20 min, the media were removed and replaced with water. At various time points, cells were imaged with a 60× objective on a TE2000‐S Nikon Eclipse using differential interference contrast (DIC) microscopy. The proportion of cells with CVs larger than ¼ the size of the cell was calculated, and > 100 cells were analyzed for each cell type at each time point. The proportion of cells with large CVs was analyzed for significant differences between the parental strains and their respective *cpnA* KO cells using a two‐proportion *z*‐test. Cells were also analyzed for viability after 22 h in water. Cells were resuspended in water at a density of 1 × 10^6^ cells per mL and incubated in a shaking incubator at 21 °C at 125 r.p.m. for 22 h. Trypan blue was added to cell samples to exclude dead cells, and samples were counted using a hemocytometer. Viability was calculated by dividing cell density after 22 h in water by initial cell density. Three trials were performed, and mean data were analyzed for significant differences between parental strains and their respective *cpnA* KO cells using a *t*‐test.

For time‐lapse imaging, cells were incubated in water for 2 h before taking DIC images every 2 s for 10 min. Thirty cells of each cell type were analyzed for how long each visible CV persisted during the 10‐min time period and the mean duration of all CVs was averaged. Mean data were analyzed for significant differences between parental strains and their respective *cpnA* KO cells using a *t*‐test.

NC4A2 and *cpnA*(NC4A2)*^−cre^* cells were transformed to express the CV marker protein, dajumin, tagged with GFP [29]. Transformed cells were incubated in water in a similar manner and imaged with a Nikon A1R CLSM every 2.2s for 5min. Twenty cells from each series of time‐lapse images were analyzed. The timing of formation and expulsion of each CV was determined, and the area of each CV in each image was measured using imagej  (Research Services Branch, National Institute of Mental Health, Bethesda, MD, USA). Mean data were analyzed for significant differences between NC4A2 and *cpnA*(NC4A2)*^−cre^* cells using a *t*‐test.

### Bead macropinocytosis assay

To measure macropinocytosis of fluorescent beads, NC4A2 and *cpnA* KO cells were incubated with small beads (0.04‐µm dark red FluoSpheres carboxylate‐modified microspheres; Thermo Fisher, 1795452, Waltman, MA, USA) in shaking suspension and the mean cell fluorescence was measured using flow cytometry. *Dictyostelium* cells were harvested by centrifugation at 437 ***g*** for 5 min at 4 °C and resuspended at a concentration of 2 × 10^6^ cells per mL. Cells were incubated with 5.69 × 10^14^ beads per mL in 50‐mL flasks containing 2.5 mL of HL‐5 and allowed to macropinocytose for 30 min at 180 r.p.m. At 5‐min time points, 100 µL of cells was transferred from the flask into a microcentrifuge tube and cells were washed free of unbound beads with fresh media by centrifugation at 437 ***g*** for 5 min at 4 °C three times. The pellet was resuspended in 3.7% formaldehyde in Sorensen’s Buffer (0.2 m sodium phosphate, pH 6.5) for flow cytometry and washed two times in Sorensen’s Buffer. Fixed cells were analyzed on a Beckman Coulter CytoFLEX Flow Cytometer with the 638‐nm laser (Brea, CA, USA). Cells without beads were used to gate for cells. Gated events were analyzed for mean cell fluorescence, and 10 000 gated events were recorded at each time interval. Mean cell fluorescence data were normalized to the average mean fluorescence at all time points for the NC4A2 cells. Normalized data from seven trials of each cell type were averaged and analyzed for significant differences at each time point between NC4A2 and *cpnA* KO cells using repeated‐measures ANOVA and *post hoc* Tukey’s comparisons.

Cells were incubated with beads as previously described. Cells at each time point were plated on glass coverslips and allowed to settle for 15 min. Cells were then fixed in 3.7% formaldehyde in methanol for 10 min at −20 °C. Cells were washed three times with ice‐cold Sorensen’s Buffer with 5 min in between washes on coverslips. Coverslips were mounted cell side down onto glass slides with 50% glycerol in PBS. Cells were imaged with a 40× objective on a Leica DMi 8 microscope Wetzlar, Germany).

### Immunofluorescence with p80 antibody

Postlysosomes were labeled by immunofluorescence using an antibody against the endosomal membrane protein, p80 (H161, Developmental Studies Hybridoma Bank). This antibody has been shown to bind to PLs in higher concentrations compared with other endosomal vesicles and consequently is a useful tool to identify PLs [23]. To label PLs, 2 × 10^6^ cells per mL of NC4A2 and *cpnA* KO cells were placed on coverslips and flattened with agarose overlays [30] and fixed in 3.2% formaldehyde in methanol for 10 min at −20 °C. Cells were blocked in 300 mm glycine, 0.05% Triton X‐100 in PBS for 30 min, and incubated with the p80 primary antibody (1 : 200) overnight at 4 °C. The next day, cells were washed three times with 0.05% Triton X‐100 in PBS and incubated with a secondary goat anti‐mouse antibody conjugated to Alexa Fluor 488 (1 : 1000) (Fisher Scientific, A‐11001) for 1 h and washed again three times with 0.05% Triton X‐100 in PBS. Coverslips were mounted on slides with 100 mg·mL^−1^ DABCO and 50% glycerol in PBS. Cells were imaged with fluorescence microscopy using a TE2000‐S Nikon Eclipse. Cells were also imaged using a Nikon A1R CLSM, and max intensity projections were generated in NIS‐elements. The diameter of > 150 PLs from each cell type from two trials was measured using imagej. Mean diameter data were analyzed for significant differences between parental strains and their respective *cpnA* KO cells using a *t*‐test. The number of PLs per cell was counted in > 300 cells, and the data from five trials were combined. Proportions of PLs per cell data were analyzed for significant differences between the parental strains and their respective *cpnA* KO cells using a two‐proportion *z*‐test.

For imaging actin filaments, cells were colabeled with p80 antibody and rhodamine phalloidin. Cells were fixed and labeled with p80 antibody as described above, and then additionally stained with 100 nm rhodamine phalloidin (Cytoskeleton, PHDR1). The number of PLs and the number of PLs coated in a complete coat of actin were counted for each cell. Data from three trials were combined (*n* > 300 cells), and proportion data were analyzed for significant differences between the *cpnA* KO cells and parental cells using a two‐proportion *z*‐test.

### Dextran endocytosis pulse‐chase assay

The biogenesis of PLs resulting from macropinocytosis was observed in a pulse‐chase experiment. Parental NC4A2 and the respective *cpnA* KO cells were placed on glass‐bottom dishes and incubated in HL‐5 containing 0.4 mg·mL^−1^ fluorescein isothiocyanate (FITC)‐dextran (BioChemika, 46945) and 4 mg·mL^−1^ tetramethylrhodamine (TRITC)‐dextran (BioChemika, 46945) for 17 min. Cells were washed with fresh HL‐5 twice and imaged at 5‐min increments for 2 h starting at 25 min. The number and area of PLs per cell at each time point were measured by colocalization of red and green signal defined with an imagej colocalization plugin (https://imagej.nih.gov/ij/plugins/colocalization.html). To calculate area of PLs per cell, the colocalization area in each image was divided by number of cells. The colocalization area in each image was also divided by the number of PLs in each image to estimate the size of PLs. The estimated area of PLs was used to estimate the diameter of PLs at each time point. Three trials for each cell type were performed and averaged. Differences between cell types at each time point were analyzed using a repeated‐measures ANOVA and *post hoc* Tukey’s comparisons.

## Results

### Creation of AX4 cpnA null mutants

Cells lacking the *cpnA* gene using the axenic strain, NC4A2, were made previously by replacing the *cpnA* gene with the blasticidin resistance (*bsr*) gene [12, 15]. Because various *Dictyostelium* axenic laboratory strains have been shown to carry different mutations and duplications [31], we wanted to test whether removing the *cpnA* gene from another axenic laboratory strain, AX4, would result in a similar phenotype. The same two *cpnA* KO plasmids that were used to make the *cpnA* KO cell lines in NC4A2 cells were electroporated into AX4 cells. Electroporated cells were plated in 96‐well plates and treated with blasticidin. Transformed cells resistant to blasticidin were further screened with polymerase chain reaction and western blot (Fig. 1). The nomenclature used in this study to describe the *cpnA* KO cells made previously in NC4A2 cells and those made in AX4 cells is shown in Fig. 1A. A 1008‐bp region within the *cpnA* open reading frame was amplified by PCR from parental cell lines (AX4 and NC4A2), but not *cpnA* KO cells, indicating successful replacement of the *cpnA* gene by the *bsr* gene (Fig. 1B). Whole‐cell samples from *cpnA* KO cell lines lacked the 66 kDa CpnA protein in western blots, while parental cells did not (Fig. 1C). Therefore, we obtained four independent *cpnA* KOs cell lines, one with and one without the loxP sites flanking the incorporated *bsr* gene, in both NC4A2 and AX4 parental axenic laboratory strains. In this study, both the NC4A2 and AX4 strains were used in the CV assays; however, only the data for NC4A2 strains are reported for the endocytosis assays. This is because we found that various AX4 strains assayed behaved quite differently from each other and from the NC4A2 strains in the endocytosis assays, making it difficult to make conclusions with respect to the AX4 *cpnA* KO strains.

### *cpnA* KO cells have larger, persistent contractile vacuoles

**Fig. 2:**
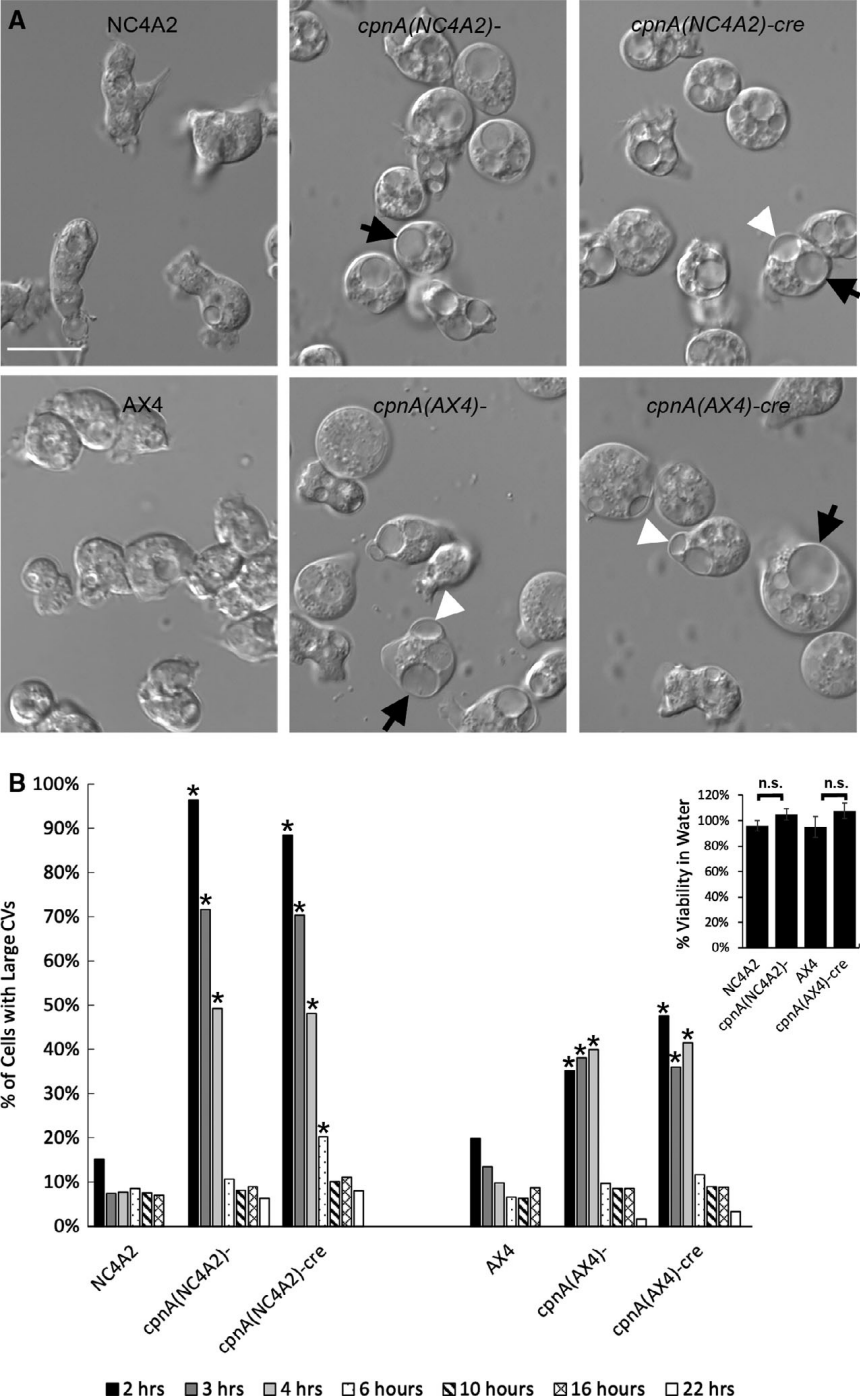
*cpnA* KO cells have large CVs. (A) Parental and *cpnA* KO cells were submerged in water for 2 h and then imaged with DIC microscopy. Scale bar = 15 µm. Black arrows indicate large CVs, and white arrowheads indicate CVs protruding from cell surface. (B) The percent of cells with CVs larger than 25% of the total cell area were counted from images taken at 2, 3, 4, 6, 10, 16, and 22 h in water. Proportion data were analyzed for significant differences between parental cells and respective *cpnA* KO cells for each time period (*n* > 100 cells) using a two‐proportion *z*‐test. * indicates significant difference (*P* < 0.05). (B inset) Cell viability in water at 22 h for parental and one corresponding *cpnA* KO cell strain was assessed. Three trials were performed and the means analyzed for significant differences between parental and respective *cpnA* KO cells using a *t*‐test. Error bars = standard error.

Previously, we showed that when *cpnA*(NC4A2)*^−^* cells undergo hypoosmotic stress, they form abnormally large CVs [12]. In this study, we used DIC microscopy to image all four of our *cpnA* KO cell lines when submerged in water and compared them to their parental strains (Fig. 2A). *cpnA* KO cells made from AX4 cells had a similar phenotype to the *cpnA* KO cells made from NC4A2 cells in that the *cpnA* KO cells had unusually large CVs (Fig. 2A, black arrows), and some of the CVs of *cpnA* KO cells appeared to be protruding from the cell surface (Fig. 2A, white arrowheads). Large and/or protruding CVs were not as common in the parental cells.

To quantify the large CV phenotype, we determined the proportion of cells with CVs greater than one‐fourth the size of the whole cell from DIC images at different time points after placing them in water (Fig. 2B). The parental NC4A2 and AX4 cells behaved similarly with 10–20% of cells having large CVs after 2 h. The percent of cells with large CVs decreased over time, and at 22 h, there were no cells with large CVs in the parental strains. The *cpnA* KO cells from the NC4A2 strain were significantly different from the NC4A2 cells with over 90% of cells having large CVs at 2 h. The large CV phenotype was also seen in the *cpnA* KO cells from the AX4 strain, but the phenotype was not as pronounced as in the NC4A2 cells, in that only ~ 40% of cells had the large CV phenotype. As previously described for *cpnA*(NC4A2)*^−^* cells, the AX4 *cpnA* KO cells were also able to adapt to the hypoosmotic conditions over time and only a small percentage of cells had large CVs at 22 h (Fig. 2B). Neither the parental nor the *cpnA* KO cells exhibited a decrease in viability after 22 h in water (Fig. 2B, inset).

**Fig. 3:**
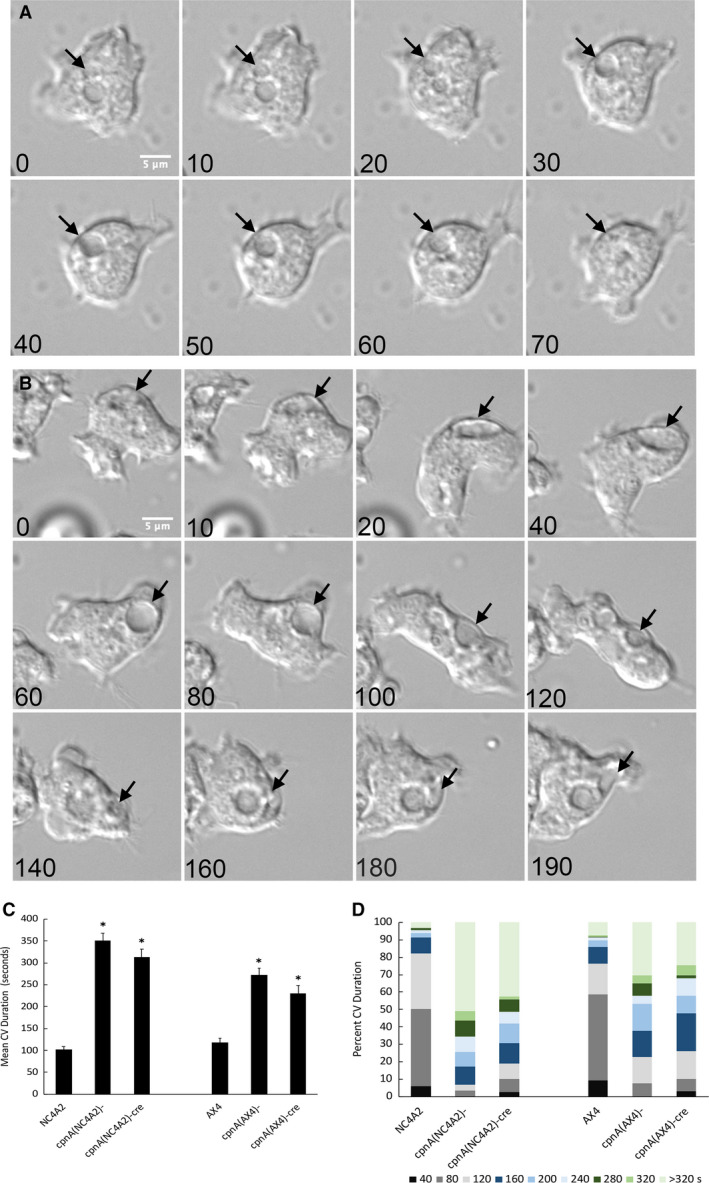
CV in *c* *pnA* KO cells persist longer. Cells were placed in water and imaged every 2 s for 10 min using DIC microscopy. Representative images of NC4A2 (A) and *cpnA*(NC4A2)*^−^* (B) cells are shown over time. Scale bar = 5 µm. The filling and expulsion of a single CV is indicated by a black arrow. Time in seconds is given in lower‐left corner of each image. (C) All CVs from 30 cells of each cell type were tracked during at 10‐min period, and the average duration time was calculated. Mean data were analyzed for significant differences between *cpnA* KO cells and parental cells with a *t*‐test. *indicates significant difference (*P* < 0.05). Error bars = standard error. (D) The same data in C were used to calculate the percentage of CVs that persisted for equal to or less than the various time categories.

To further characterize the large CV phenotype in *cpnA* KO cells, we used time‐lapse imaging to determine how long single CVs persisted before being expelled (Fig. 3). Cells were placed in water and imaged every 2 s for 10 min. Thirty cells of each cell type were analyzed, and the number of frames each CV was present in the time‐lapse images was counted. Figure 3A follows a single CV (black arrows) in an AX4 cell filling and then expelling within 70 s. Following the filling and emptying of a single CV over time in the *cpnA* KO cells was more difficult, in that the CVs seemed to often disappear and then reappear, suggesting that the CVs did not completely empty before refilling. Figure 3B follows a single CV (black arrows) in *cpnA*(AX4)*^−^* cells. The CV fills with normal timing, but becomes unusually large and persists for more than twice as long as the CV in the AX4 cell in Fig. 3A. At 140 s, the CV seems to be expelled, but then appears to refill. The average time CVs were present in NC4A2 and AX4 cells was 101 ± 7.44 s and 118 ± 9.04 s, respectively, while the CVs in the *cpnA* KO cells on average persisted for over 2–3 times longer (Fig. 3C). Similar to the less severe large CV phenotype, the CV persistence phenotype in the AX4 *cpnA* KO cells was less pronounced than in the NC4A2 *cpnA* KO cells. Not all CVs in the *cpnA* KO cells persisted longer than CVs in the parental strains (Fig. 3D). About 10% of the CVs in *cpnA* KO cells persisted for < 80 s, while 50% and 60% of NC4A2 and AX4 cells persisted for < 80 s. Likewise, only a very small percentage of CVs in the parental strains persisted for more than 320 s, while 51% of CVs in *cpnA*(NC4A2)*^−^* cells, 43% of CVs in *cpnA*(NC4A2)*^−cre^* cells, 30% of CVs in *cpnA*(AX4)*^−^* cells, and 25% of CVs in *cpnA*(AX4)*^−cre^* cells persisted for longer than 320 s (Fig. 3D).

**Fig. 4:**
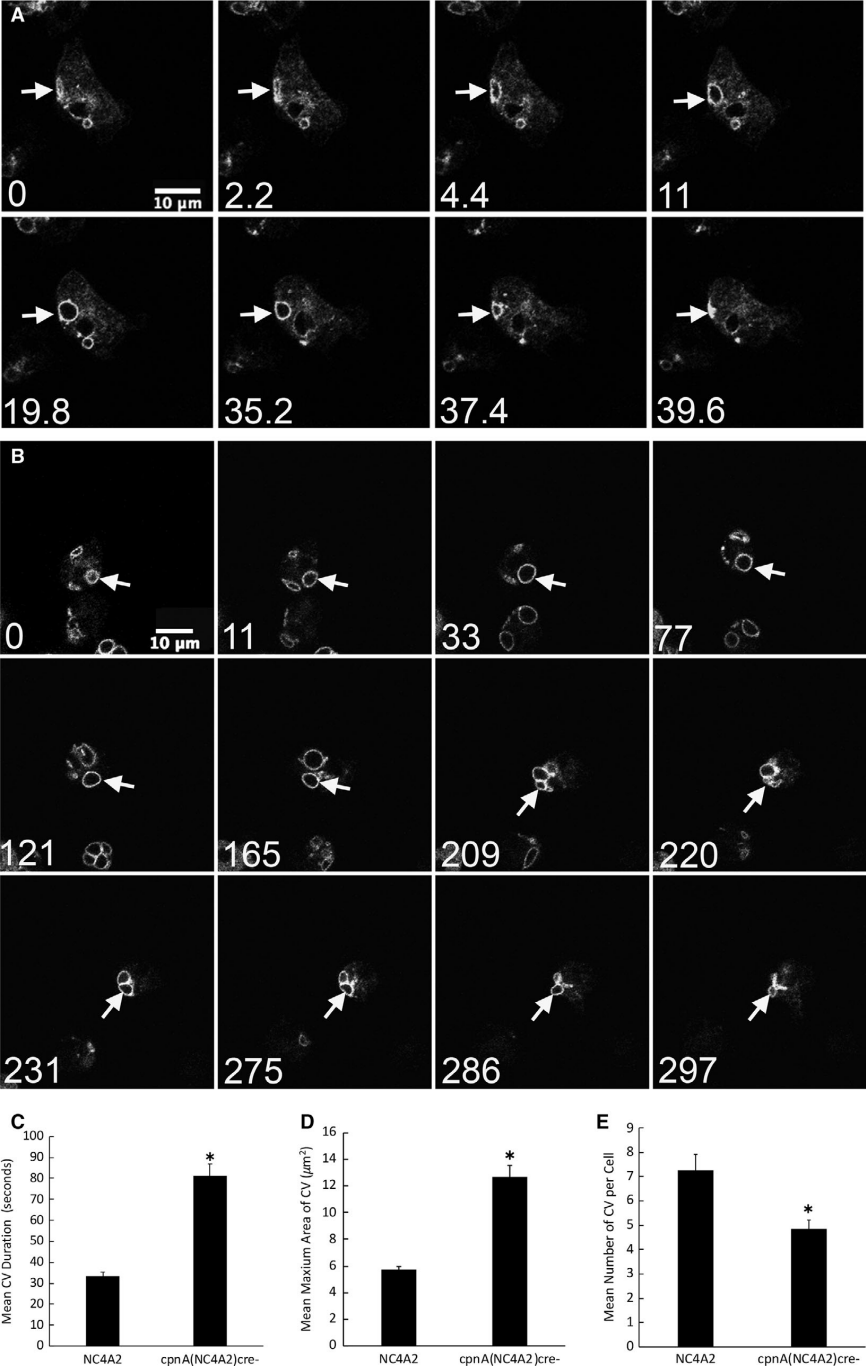
CVs visualized with GFP‐dajumin are larger and persist longer in *cpnA* KO cells. Cells expressing DAJ‐GFP were placed in water and imaged every 2.2 s for 5 min using confocal microscopy. Scale bar = 10 µm. Representative images of a single NC4A2 cell (A) and *cpnA*(NC4A2)*^−^* ^*cre*^ (B) cell are shown over time. Arrows point to a single contractile over time. Time in seconds is given in the lower left corner of each image. (C) Average CV duration. (D) Average maximum area of CVs. (E) Average number of CVs per cell. Data were analyzed from 20 cells in one time‐lapse movie for each cell type. Mean data were analyzed for significant differences between *cpnA* KO (*n* = 20) and parental cells (*n* = 20) with a *t*‐test. * indicates significant difference (*P* < 0.05). Error bars = standard error.

The CV defect was further examined in NC4A2 and *cpnA*(NC4A2)*^−cre^* cells by expressing the CV marker protein, dajumin, tagged with GFP (DAJ‐GFP) in these cell lines. Dajumin is a glycoprotein known to associate with CV membranes [29]. Cells were placed in water and imaged every 2.2 s for 5 min using confocal microscopy (Fig. 4). The CVs of 20 cells were followed, and the diameter of each CV was measured at each time point. In Fig. 4A a single CV (white arrow) in a NC4A2 cell is filled and then is expelled within 40 s. In Fig. 4B a single CV (white arrow) in a *cpnA*(NC4A2)*^−cre^* cell is filled, appears to get smaller at 220 s, but then expands again before getting smaller again. On average, the DAJ‐GFP‐labeled CVs in NC4A2 cells persisted for 33.5 ± 1.8 s, while DAJ‐GFP‐labeled CVs in *cpnA*(NC4A2)*^−cre^* cells persisted for 81.1 ± 5.9 s (Fig. 4C). The average single CV duration time was much less than what was calculated from the time‐lapse DIC images. This is likely due to the fact that the DAJ‐GFP marker allowed us to follow smaller CVs and CVs not visible in the DIC images. In addition, cells were only imaged for 5 min instead of 10 min. However, similar to the DIC image data, on average the CVs of *cpnA*(NC4A2)*^−cre^* cells persisted 2.5 times longer than the CVs of the parental NC4A2 cells. The average maximum area of CVs labeled with GFP‐DAJ in NC4A2 cells was 5.7 ± 0.3 µm^2^, while the average maximum area of CVs labeled with GFP‐DAJ in *cpnA*(NC4A2)*^−cre^* cells was 12.7 ± 0.9 µm^2^ (Fig. 4D). The number of CVs per cell formed over 5 min was 7.2 ± 0.65 for NC4A2 cells and 4.9 ± 0.3 for *cpnA*(NC4A2)*^−cre^* cells (Fig. 4E). These data indicate that *cpnA* KO cells have fewer CVs in a given time period and these CVs are larger and persist for longer periods of time. Overall, this suggests that CpnA may play a role in regulating the size of CVs and perhaps acts as a positive regulator of CV exocytosis.

### Cargo is transported through the endolysosomal pathway faster in *cpnA* KO cells

**Fig. 5:**
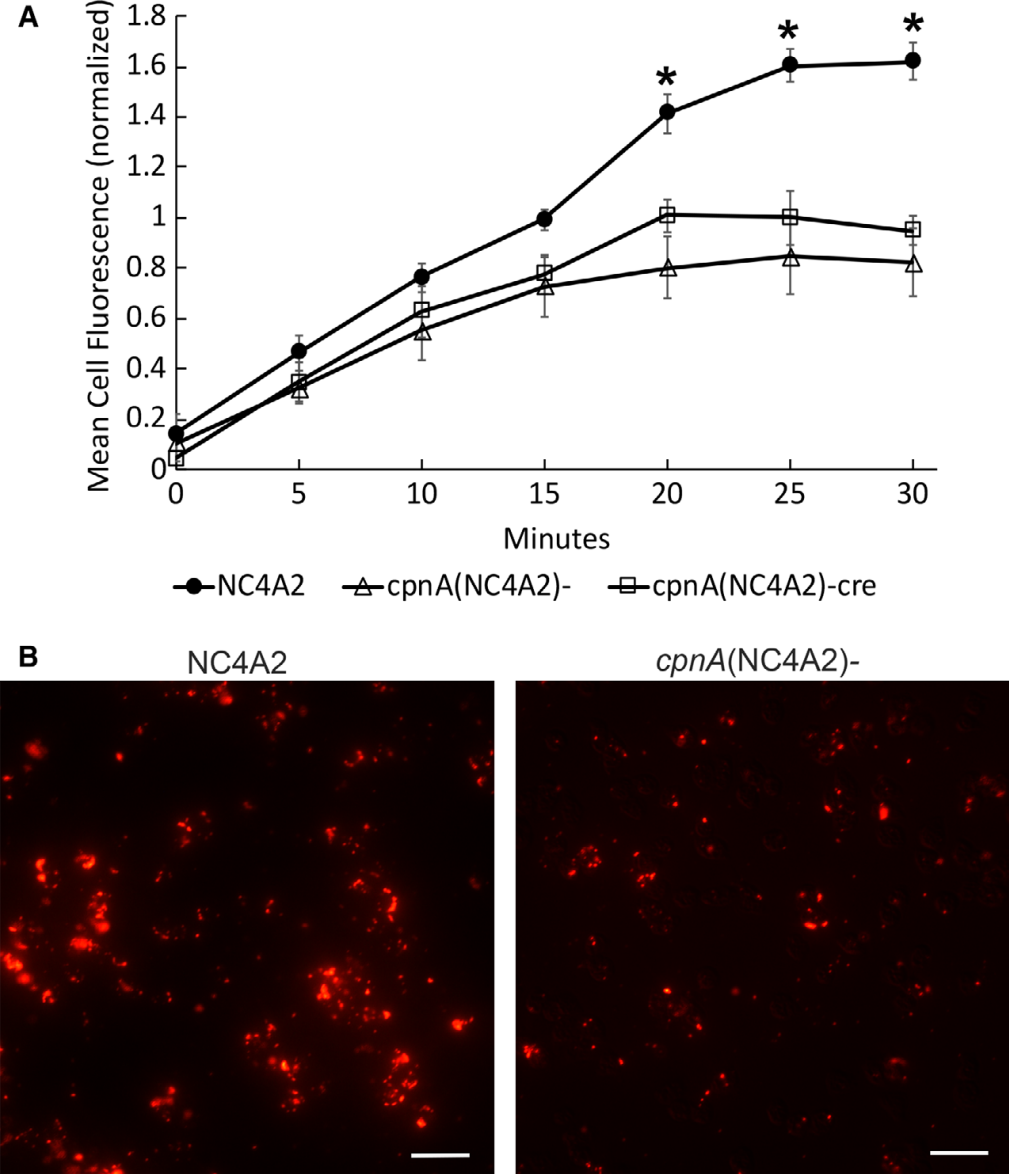
Material moves through the endolysosomal pathway faster in *cpnA* KO cells. (A) NC4A2 and *cpnA* KO cells were allowed to endocytose 0.04‐µm beads in a shaking suspension for 30 min. Cell samples were taken out every 5 min, washed with Sorensen’s Buffer, fixed using 3.7% paraformaldehyde, and analyzed using flow cytometry. Experiments were performed seven times for each cell line, and fluorescence at each time point was normalized to the average fluorescence over all time points for the NC4A2 cells within each trial. Differences among the cell types at each time point were analyzed with a repeated‐measures ANOVA and *post hoc* Tukey’s comparisons. *indicates *cpnA* KO cells are significantly different (*P* < 0.05) from NC4A2 cells. Error bars = standard error. (B) NC4A2 and *cpnA(*NC4A2*)^−^* cells were fixed on coverslips with 3.7% paraformaldehyde in methanol after 30 min in a shaking suspension with beads. Cells were imaged on a Leica DMi 8 microscope. Scale bar = 20 µm.

*Dictyostelium* take up nutrients mainly by phagocytosis of bacteria and yeast. However, axenic laboratory strains have been mutated so that they can thrive by taking up fluid nutrients by macropinocytosis. In a previous study, we performed macropinocytosis assays using a fluorimeter to measure the uptake of TRITC‐dextran. We found little difference in the rates of uptake between NC4A2 and *cpnA*(NC4A2)*^−^* cells [12]. However, in this study, we assayed macropinocytosis by using flow cytometry to measure the uptake of 0.04‐µm fluorescently labeled beads. NC4A2, *cpnA*(NC4A2)*^−^*, and *cpnA*(NC4A2)*^−cre^* cells were incubated with the beads for 30 min, and the amount of fluorescence inside the cells was measured at 5‐min time points. Initially, *cpnA* KO cells appeared to take up beads at the same rate as the parental cells, as seen previously. However, at the later time points, the amount of fluorescence in the *cpnA* KO cells began to plateau earlier than in the parental cells (Fig. 5A). When cells were imaged at the later time points, images indicated that *cpnA* KO cells had fewer organelles containing the fluorescent beads and the organelles appeared to be smaller in the *cpnA* KO cells than the parental cells (Fig. 5B). These data suggest that *cpnA* KO cells were able to take up the beads similarly to the parental cells, but that the beads moved through the endolysosomal system faster in the *cpnA* KO cells than the parental cells. The decrease in the number of large organelles containing beads in the *cpnA* KO cells suggested a problem with the formation of PLs, the neutral and final organelle of the endolysosomal pathway that is typically twice the size of lysosomes [23].

### *cpnA* KO cells have fewer and smaller postlysosomes

**Fig. 6:**
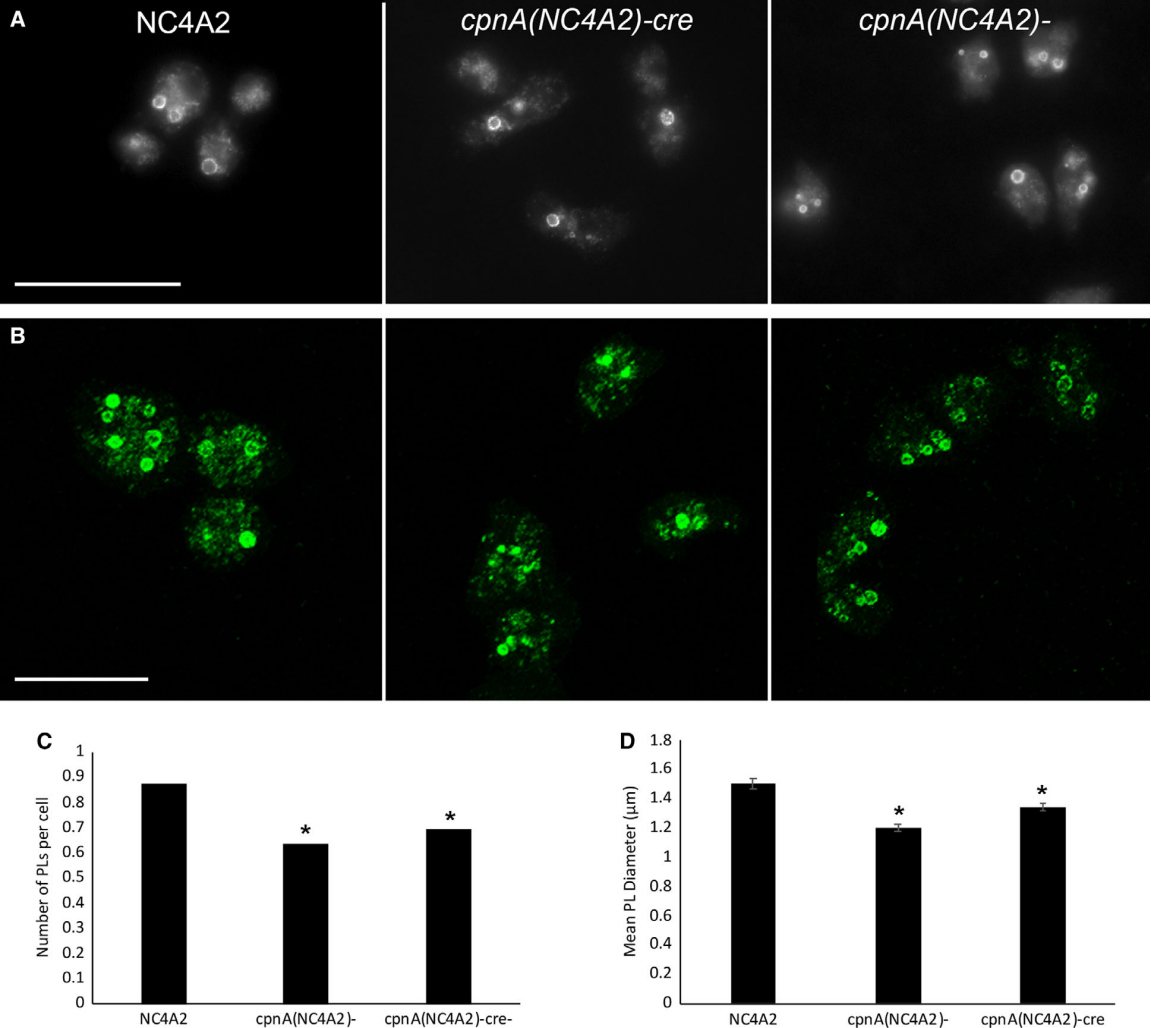
*cpnA* KO cells have fewer and smaller PLs. (A) Epifluorescence images of cells labeled with p80 antibody, which is enriched on PL membranes, and goat anti‐mouse antibody conjugated to Alexa Fluor 488. Scale bar = 30 µm. (B) Confocal z‐series max projections of cells labeled with p80 antibody. Scale bar = 25 µm. (C) Number of PLs per cell (> 300) for each cell type. Data from five trials were combined, and proportion data were analyzed for significant differences between the *cpnA* KO cells and parental cells using a two‐proportion *z*‐test. (D) Average diameter of > 150 PLs for each cell type. Mean data from two trials were analyzed for significant differences between *cpnA* KO cells and parental cells with a *t*‐test. *indicates significant difference (*P* < 0.05). Error bars = standard error.

To identify PLs and measure their size, we used an antibody to p80, a putative copper transporter present on membranes throughout the entire endolysosomal system, but enriched on the PL membrane [32]. p80 labeling in *cpnA* KO and parental cells was imaged with epifluorescence. In these images, PLs were defined as brightly labeled vesicles (Fig. 6A). To capture all the PL within cells, we imaged p80‐labeled cells with confocal microscopy and created max intensity projections (Fig. 6B). We then used imagej to count the number of PLs and measure the diameter of each PL. The *cpnA* KO cells had fewer PLs per cell (0.65 and 0.70) compared with NC4A2 (0.87) (Fig. 6C). The PLs of *cpnA* KO cells were also smaller. The average diameter of the PLs was 1.50 ± 0.03 µm in NC4A2 cells, while the average diameter of PLs in the *cpnA* KO cells was smaller: 1.20 ± 0.03 µm for *cpnA*(NC4A2)*^−^* cells and 1.34 ± 0.03 µm for *cpnA*(NC4A2)*^−cre^* (Fig. 6D). These results suggest that *cpnA* KO cells are defective in regulating PL size and that CpnA may play a role in the maturation of PLs.

### Postlysosomes are exocytosed before reaching full size in *cpnA* KO cells

**Fig. 7:**
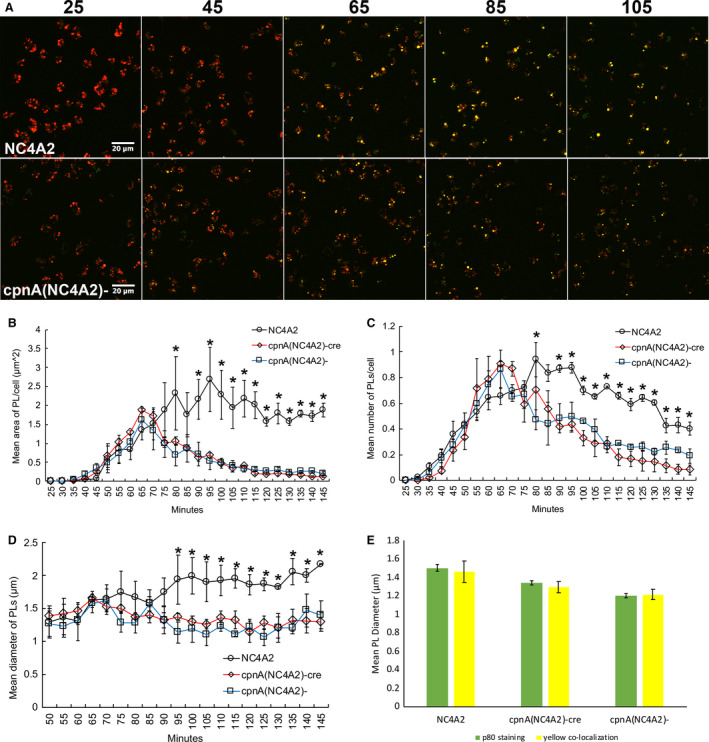
PLs are exocytosed before reaching full size in *cpnA* KO cells. Cells were incubated with TRITC and FITC‐labeled dextran for 15 min and then washed. Cells were imaged with confocal microscopy every 5 min for 2 h starting at 25 min after incubation with dextran. imagej was used to quantify the red and green colocalization (PL) area in each image. Scale bar = 20 µm. Data from three trials were averaged. Error bars = standard error. (A) Representative images from various time points after incubation with dextrans for NC4A2 and *cpnA*(NC4A2)*^−^* cells. Minutes are shown above images. (B) Average PL area per cell over time. (C) Average number of PLs per cell over time. (D) Average PL diameter over time. Differences among the cell types at each time point were analyzed with a repeated‐measures ANOVA and *post hoc* Tukey’s comparisons. *indicates both *cpnA* KO cells are significantly different (*P* < 0.05) from parental cells, but not from each other. Error bars = standard error. (E) A comparison between average PL diameter observed using p80 staining at a fixed time and average PL diameter observed using labeled dextrans from averaging diameters at each time point.

To analyze PL biogenesis and maturation in live cells, we performed a pulse‐chase endocytosis assay. Cells were incubated with indigestible dextran conjugated to two different fluorophores, TRITC and FITC. TRITC‐dextran is pH‐independent and therefore fluorescent throughout the entire endolysosomal system, while FITC‐dextran is only fluorescent at a neutral pH. By feeding cells a combination of the two dextrans, the pH of vesicles is labeled in live cells and allows for the distinction between acidic and neutral compartments in the endolysosomal pathway. Red represents acidic endosomes and lysosomes, while yellow, because of the presence of both the red and green signals, labels neutral PLs (Fig. 7A). Cells were incubated with both dextrans for 17 min, then the dextrans were washed from the cells, and the cells were imaged every 5 min with confocal microscopy starting at 25 min. imagej was used to measure the area of red and green colocalization in each image. In addition, the number of cells in each image and the number of postlysosomes defined by red and green colocalization (yellow) in each image were counted. Figure 7B shows the total area of red and green colocalization in each image divided by the number of cells to give an estimation of the mean area of PLs per cell. These data revealed that the PL area per cell in *cpnA* KO cells is not different from parental cells early on but then differs drastically from the parental NC4A2 cells after 70 min. The total PL area per cell peaked at 65 min in the *cpnA* KO cells and then rapidly decreased, while PL area per cell in the parental cells peaked at 95 min and then remained steady (Fig. 7B).

Counting the number of PLs per cell revealed that *cpnA* KO cells and the parental NC4A2 cells form PLs with similar timing. However, the number of PLs per cell peaked 10–15 min earlier in the *cpnA* KO cells than the parental cells and the PLs in the *cpnA* KO cells were nearly absent by 145 min (Fig. 7C). This indicates that there are fewer dextran‐labeled PLs in *cpnA* KO cells at the later time points (Fig. 7C) and suggests that the PLs are being exocytosed sooner. To estimate the size of PLs at each time point, we divided the total red and green colocalization area in each image by the total number of PLs to get an estimate of the average area of an individual PL. We then calculated the diameter from the area (Fig. 7D). These calculations showed that the PL size in *cpnA* KO cells remained fairly steady over 2 h and that the PLs did not reach the fully mature size of 2 µm in diameter. However, the PLs in the parental cells did reach the size of 2 µm in diameter by 95 min (Fig. 7D).

The diameter calculated from the colocalization data from each image was similar to the p80 data (Fig. 6C), in that the *cpnA* KO cells had smaller PLs and did not have many mature 2 µm diameter PLs. The average calculated diameter of PLs over time for the NC4A2 cells was 1.56 ± 0.11 µm, while the average calculated diameter of PL over time for the *cpnA* KOs was 1.28 ± 0.05 µm and 1.22 ± 0.05 µm for *cpnA*(NC4A2)*^−cre^* and *cpnA*(NC4A2)*^−^* cells, respectively (Fig. 7E). These data match the data obtained from measuring the diameter of p80‐labeled PLs from images taken at a single time point (Fig. 6C). In addition, the number of PLs per cell calculated from the colocalization data is similar to the number of PLs per cell data obtained with p80 labeling (Fig. 6B). The fact that these two different methods give similar results with regard to PL number and size suggests that p80 enrichment is an appropriate indicator of PLs and that we are not missing many weakly labeled PLs or counting brightly labeled lysosomes.

Taken together, these results indicate that *cpnA* KO cells are able to endocytose material, deliver the material to endosomes and lysosomes, and convert lysosomes to neutral PLs. However, the PLs in *cpnA* KO cells do not become fully mature in size and appear to be exocytosed sooner than in parental cells. Overall, this suggests that CpnA may act as a positive regulator of PL maturation and/or a negative regulator of PL exocytosis.

### *cpnA* KO cells have more postlysosomes with actin coats

**Fig. 8:**
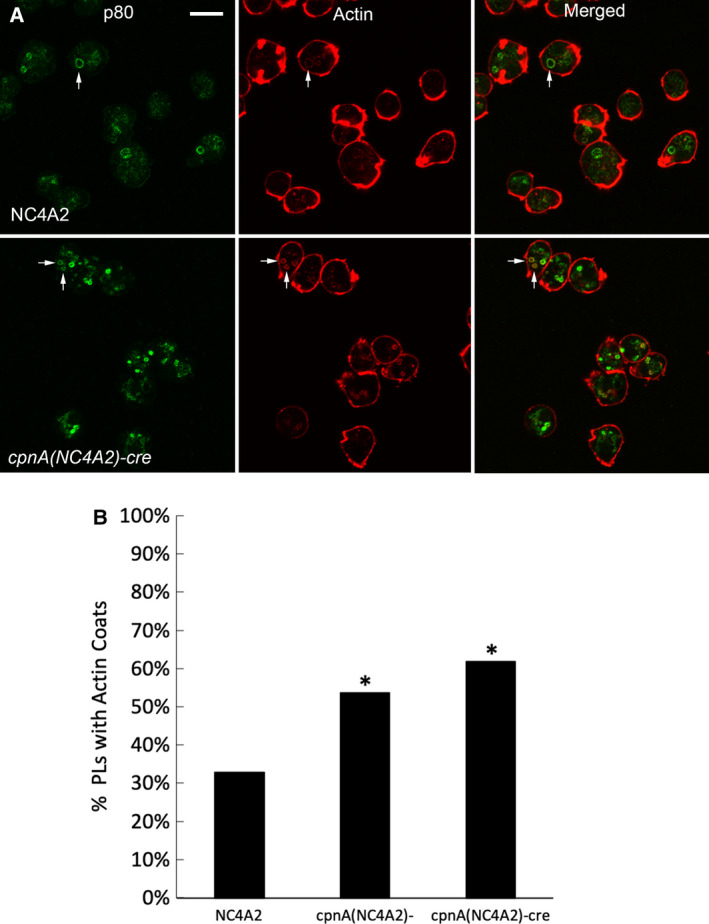
*cpnA* KO cells have more PLs with actin coats. (A) PLs were labeled with p80 antibody, and actin filaments were stained with rhodamine phalloidin. Cells were imaged with confocal microscopy. Scale bar = 10 µm. (B) Percent of PLs with actin coats for each cell type. Data from three trials were combined (*n* > 300 cells), and proportion data were analyzed for significant differences between the *cpnA* KO cells and parental cells using a two‐proportion *z*‐test. *indicates significant difference (*P* < 0.05).

Actin filaments bind to vacuolar (H^+^)‐ATPases of the lysosomal membrane and facilitate the pinching off of membrane vesicles containing the proton pumps leading to the neutralization of the lysosome into a PL [22]. After removal of vacuolar (H^+^)‐ATPases, an actin coat remains [33]. Actin filaments have been observed to associate with PLs a second time immediately before exocytosis [34]. We previously showed that CpnA can bind to actin filaments [15], and we hypothesized that the lack of CpnA may be affecting actin filament dynamics on PL membranes. Therefore, we imaged cells with p80 antibody‐labeled PLs and rhodamine phalloidin‐labeled actin filaments (Fig. 8A). The number of actin coats associated with PLs was counted in NC4A2 and *cpnA* KO cells. A larger percentage of PLs in *cpnA* KO cells had actin coats compared with PLs in NC4A2 cells (Fig. 8B). This suggests that the differences in PL maturation observed in *cpnA* KO cells may involve defects in actin filament disassembly at the PL surface.

## Discussion

CpnA’s association with endosomes, lysosomes, and CV membranes [11] led to the questions addressed in this study: whether CpnA was specifically involved in the function of these organelles. When we first characterized the defects of *cpnA*(NC4A2)*^−^* cells, we found that they made unusually large CVs when placed in water [12]. Here, we characterized the phenotype further and found that not only do *cpnA* KO cells make larger CVs, but the CVs also persist for 2–3 times longer. In addition, *cpnA* KO cells do not appear to be able to completely expel their CVs before refilling, indicating a problem with exocytosis of CVs. In a previous study, we also carried out endocytosis assays with *cpnA*(NC4A2)*^−^* cells using TRITC‐dextran and a fluorimeter to measure the amount of fluorescence released when cell samples were treated with Triton X‐100 [12]. With this method, we did not observe any differences between *cpnA*(NC4A2)*^−^* and NC4A2 cells, and the fluorescence plateaued later for both cell types. However, in the current study, we incubated cells with small fluorescent beads and then used flow cytometry to measure fluorescence in individual cells. Flow cytometry allows for the measurement of fluorescence associated with individual cells and therefore is a more precise way to measure endocytosis compared with lysing cells and measuring released fluorescence. Using flow cytometry, we found that the *cpnA* KO cells endocytosed the beads at normal rates, but reached a plateau sooner, compared with NC4A2 cells. Further investigation of *cpnA* KO cells indicated that they have fewer PLs and that PLs do not reach the fully mature size. Pulse‐chase assays suggested that PLs are exocytosed sooner as compared to parental cells.

Exocytosis involves multiple events including targeting, tethering, docking, and fusion of vesicles with the plasma membrane. CpnA could potentially be involved in any of these steps by interacting with CV or PL membranes and/or the plasma membrane and any number of proteins. Interestingly, the main findings of this study indicate that CpnA plays opposite roles in CV exocytosis and PL exocytosis. In *cpnA* KO cells, CVs become larger than normal and are not exocytosed efficiently, while PLs are smaller than normal and appear to be exocytosed prematurely. These results suggest that CpnA is a positive regulator of CV exocytosis and a negative regulator of PL exocytosis. Alternatively, as discussed below, CpnA may act upstream of PL exocytosis and function as a positive regulator of lysosomal fusion events necessary for the maturation of PLs.

Because CpnA was previously shown to bind actin filaments [15] and actin filaments are known to associate with PLs, we used rhodamine phalloidin staining to determine whether actin filament association is different in *cpnA* KO cells. We found that *cpnA* KO cells had a higher percentage of PLs with actin coats than parental cells, suggesting that CpnA may regulate the dissociation of actin filaments from PLs. Actin filaments associate with PL at two different steps: during the recycling of the vacuolar (H^+^)‐ATPases via a WASP and SCAR homolog known as WASH [22] leading to the neutralization of the lysosome into a PL and a second time immediately before exocytosis [34]. Because PLs of *cpnA* KO cells become neutral with similar timing as parental cells, the removal of the proton pumps does not appear to be obstructed, suggesting the increase in PL actin coats in *cpnA* KO cells does not impede proton‐pump recycling. In fact, membrane recycling might be enhanced in *cpnA* KO cells leading to smaller PLs and as a result, their premature disappearance. A study characterizing phagosomal proteins reported that CpnA and CpnB are reduced on phagosomes in WASH mutants [35], indicating that CpnA binding to lysosomal membranes is dependent on WASH and therefore may occur after the formation of actin filaments on PL membranes.

Drengk *et al*. [36] created a hybrid protein of vacuolin and cofilin to specifically cause the depolymerization of actin coats on maturing PLs. As a consequence, PLs lost their actin coats and were found in clusters. They hypothesized that actin coats prevented aggregation and thereby lysosomal fusion events. If actin coats prevent lysosomal fusion, then the increase in actin coats observed in *cpnA* KO cells could be preventing fusion events necessary for PL maturation and cause PLs to remain small. Therefore, it is possible that instead of specifically acting as a negative regulator of exocytosis, CpnA may act as a positive regulator of lysosomal fusion events necessary for PL maturation. PLs may be exocytosed earlier in *cpnA* KO cells because they are bypassing PL fusion events that lead to the larger PL size.

CpnA may also play a role in the interaction or regulation between CV membranes and actin filaments. Once a CV is docked to the plasma membrane, actin filaments appear to form around the CV and provide structural support to the CV during expulsion [37]. When cells are treated with the actin‐depolymerizing drug, latrunculin, CVs collapse [37], suggesting actin filaments are necessary for CV structural support. In the absence of CpnA, actin filament dynamics may be altered so that CVs are not properly supported leading to problems in regulating size and expulsion.

Copine‐6, a mammalian brain‐specific copine, has recently been reported to interact with synaptobrevin, a synaptic vesicle and SNARE protein, and to inhibit spontaneous calcium‐evoked neurotransmitter release in mouse hippocampal neurons [10]. In this case, Copine‐6 is acting as a negative regulator of exocytosis. In the future, our experiments will focus on investigating actin filament dynamics in *cpnA* KO cells during the endolysosomal pathway and determining whether CpnA interacts with membrane fusion machinery like the SNARE complex.

## Conflict of interest

The authors declare no conflict of interest.

## Author contributions

EW and AI performed the experiments and acquired the data. EW, AI, and CD analyzed and interpreted the data, and prepared the manuscript. CD designed, supervised, and provided funding for the project. All authors have read and approved the final manuscript.
